# pH Affects the Spontaneous Formation of H_2_O_2_ at the Air–Water Interfaces

**DOI:** 10.1021/jacs.4c07356

**Published:** 2024-09-16

**Authors:** Maria Angelaki, Jill d’Erceville, D. James Donaldson, Christian George

**Affiliations:** † Universite Claude Bernard Lyon 1, CNRS, IRCELYON, UMR 5256, Villeurbanne, F-69100, France; ‡ Department of Chemistry, University of Toronto, 80 George Street, Toronto, Ontario, Canada M5S 3H6; § Department of Physical and Environmental Sciences, University of Toronto Scarborough, 1265 Military Trail, Toronto, ON, Canada M1C 1A4

## Abstract

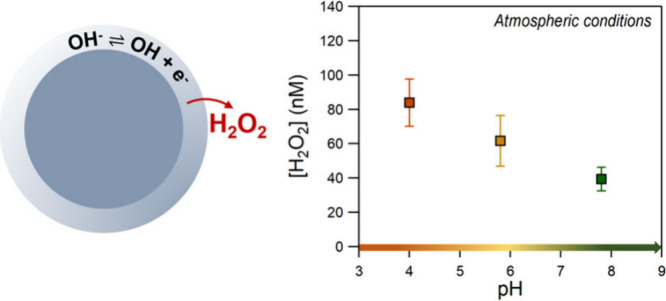

Recent studies have shown that the air–water interface of aqueous microdroplets is a source of OH radicals and hydrogen peroxide in the atmosphere. Several parameters such as droplet size, salt, and organic content have been suggested to play key roles in the formation of these oxidants. In this study, we focus on the effect of acidity on the spontaneous interfacial hydrogen peroxide formation of salt-containing droplets. Na_2_SO_4_, NaCl, and NaBr bulk solutions, at the range of pH 4 to 9.5, were nebulized, using ultra high-purity N_2_/O_2_ (80%/20%), and H_2_O_2_ was measured in the collected droplets. All of the experiments were performed in *T* = 292 ± 1 K and humidity levels of 90 ± 2%. For Na_2_SO_4_ and NaCl, the H_2_O_2_ concentration was increased by ∼40% under alkaline conditions, suggesting that OH^–^ enriched environments promote its production. When CO_2_ was added in the ultrapure air, H_2_O_2_ was observed to be lower at higher pH. This suggests that dissolved CO_2_ can initiate reactions with OH radicals and electrons, impacting the interfacial H_2_O_2_ production. H_2_O_2_ formation in NaBr droplets did not display any dependence on the pH or the bath gas, showing that secondary reactions occur at the interface in the presence of Br^–^, which acts as an efficient interfacial source of electrons.

The air–water interface of small droplets exhibits high reactivity that can lead to the acceleration of some reactions and promotes others which are thermodynamically unfavored in the bulk.^1−4^ This unique interfacial property has been attributed to the dynamical solvation behavior of solutes there that lead to large fluctuations of the external electrostatic potential,^5−7^ as well as the presence of an intrinsic local electric field.^8^

Near the air–water interface of aqueous droplets, water’s autoionization into hydronium cations (H^+^) and hydroxide anions (OH^–^) can lead to the spontaneous formation of OH radicals, solvated electrons, and H atoms (R1, R2), due to these electrostatic forces.^9−12^ Several studies have shown that hydrogen peroxide (H_2_O_2_) is formed, most probably via the mechanistic scheme described by reactions R3–R7. Two OH radicals can recombine to form H_2_O_2_ (R3).^10,11,13−16^ Solvated electrons in the presence of O_2_ can form HO_2_ radicals (R4, R5) that can lead to H_2_O_2_ production either via self-recombination (R6) and/or reaction with H atoms (R7), adding a second pathway to its formation.^12,16^
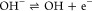
1
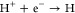
2
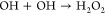
3
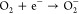
4
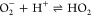
5
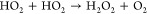
6
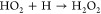
7

Interfacial H_2_O_2_ production has been studied as a function of droplet size by Lee et al.; small droplets favor its formation due to their higher surface-to-volume ratio.^13^ Mehrgardi et al. and Mofidfar et al. showed that low nebulization flow rates enhance the H_2_O_2_ formation, as it results in higher droplets’ lifetimes in the reactor, which leads to water evaporation.^15,17^ Mofidfar et al. also found that peroxide formation decreases at high humidity levels, i.e., 95% due to the water droplets’ dilution.^17^ Recently, we showed that halides such as Br^–^ and I^–^ enhance or suppress, respectively, H_2_O_2_ formation near the interfacial region, while ions like SO_4_^2–^ and Cl^–^ do not affect the overall process.^16^

There is still limited knowledge on how droplets’ pH can affect spontaneous H_2_O_2_ formation. Bulk acidity of aerosols varies from 0 to 10 depending the origin, size, chemical composition, and aging processes.^18,19^ In contrast, interfacial pH is matter of intense debate and certainly requires further investigation.^20−22^ Recent simulations have shown that the interface might be very slightly more acidic than the bulk, but not super acidic nor alkaline.^23^ Hence, we investigated the effect of (bulk) pH on interfacial H_2_O_2_ production occurring in Na_2_SO_4_-, NaCl-, and NaBr-containing droplets.

Aqueous nanodroplets were generated by nebulizing salt-containing solutions and introduced into a flow-tube reactor. The droplets were collected (after a reaction time of 10 s) as a bulk liquid phase, and the H_2_O_2_ produced in them was measured off-line, using a H_2_O_2_ analyzer (detailed experimental description in Text S1–S2, Figures S1–S3). Bulk pH values were set between 4 and 9.5 due to limitations induced by the H_2_O_2_ monitor, as at lower or higher pH values, the catalase and other reagents used in the detection are reported to be destroyed.^24,25^ This was confirmed via control experiments (Text S3 and Figure S4).

Initially, experiments were performed using Na_2_SO_4_ and NaCl solutions in a humidified 80:20 mix of high-purity (UHP) N_2_/O_2_ bath gas. Figure 1 shows the pH measurements of the bulk and collected droplets. The values were identical for all salts studied here. For both salts, the pH of the collected droplets was measured to be the same as for the bulk, within experimental uncertainty. The results of the H_2_O_2_ measurements produced in Na_2_SO_4_ and NaCl droplets are presented in Figure 2 (see Table S1). The error bars show the 2σ uncertainty and include the estimated systematic uncertainties.

**Figure 1:**
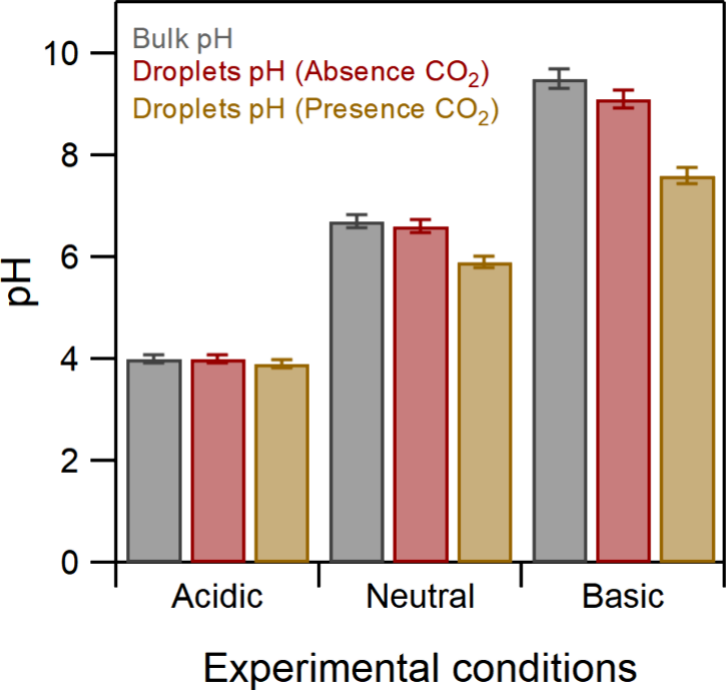
pH measurements of bulk solutions and collected droplets in 80:20 N_2_/O_2_ and compressed air.

**Figure 2:**
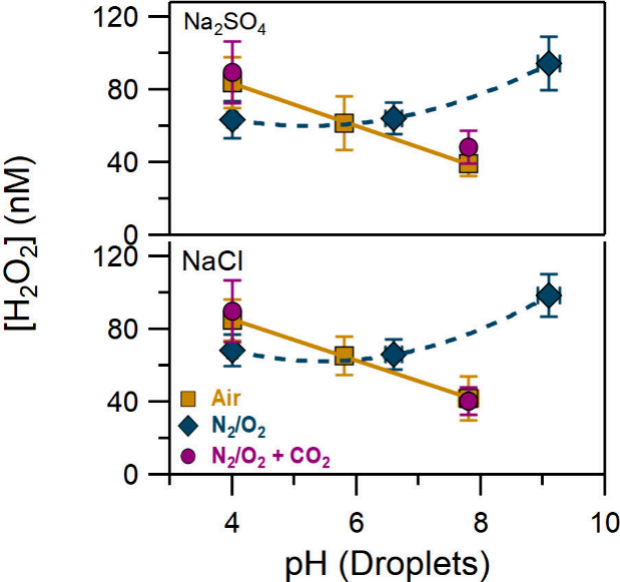
H_2_O_2_ produced in Na_2_SO_4_ and NaCl droplets (pH = 4–9.5), in N_2_/O_2_, compressed air, and N_2_/O_2_ with additional flow of CO_2_ (see legend).

For both salts, in alkaline conditions H_2_O_2_ production was increased by 40% compared to acidic conditions, indicating that the OH^–^ enriched environment promotes oxidant formation. As the reaction that initiates the chemistry is proposed to be driven by the hydroxide anion (R1), a higher concentration of OH^–^ would lead to a higher production of OH radicals and electrons and therefore H_2_O_2_. However, considering that the OH^–^ concentration was increased over 6 orders of magnitude in our measurements, the production of H_2_O_2_ did not display a strong dependence on the hydroxide concentration, suggesting that it is not proportional to [OH^–^]. Recently, it has been suggested by de la Puente et al. that interfaces are enriched with hydronium cations, while hydroxide ions are mainly underneath the interface.^23^ Tse et al. also showed that hydroxide ion is repelled from the air–water interface.^26^ Given that, the fraction of interfacial OH^–^ may be not the same as that in the bulk, in accord with our experimental observations.

To investigate these interfacial reactions under atmospherically relevant conditions, the same experiments were also performed using compressed air, which contains ambient amounts of CO_2_. Altering the bath gas gave significant differences compared to our observations with a pure N_2_/O_2_ mix. In the presence of CO_2_, H_2_O_2_ production is decreased under alkaline conditions (i.e., the opposite trend to that seen in the absence of carbon dioxide, Figure 2). In addition, using compressed air, the pH of the collected droplets generated from bulk solutions of pH 6.7 and 9.5 was decreased by 12% and 20%, respectively, while for acidic conditions, it remained stable within ±2% (Figure 1). Since the major difference between the two different experimental conditions is the presence of CO_2_ in the compressed air, we attribute the decrease of the pH seen in Figure 1 to the acidification of the droplets by dissolved carbon dioxide. To demonstrate that CO_2_ is responsible for the trend observed in Figure 2, additional experiments were performed using a mixture of UHP N_2_/O_2_ and UHP CO_2_ (Figure 2). CO_2_ concentration was set at 400 ppm, simulating atmospheric levels. The addition of CO_2_ in the N_2_/O_2_ mixture led to results identical to those obtained using compressed air, verifying that the presence of CO_2_ alters the chemistry.

CO_2_ dissolution is favored in alkaline conditions as the effective Henry’s Law coefficient is higher by 4 orders of magnitude compared to acidic environment (Text S4, Figure S5). In complete agreement with our observations, Cohen et al. reported a decrease of pH of alkaline levitated droplets due to CO_2_ uptake.^27^ Li et al.^28^ have also reported that spraying alkaline solutions of phenolphthalein in an air environment gave rise to decoloration of the droplets due to CO_2_ dissolution.

The results displayed in Figure 2 indicate that under neutral conditions, H_2_O_2_ production was not affected by a change of the bath gas, while in acidic conditions we observed a small increase in the presence of CO_2_. However, in alkaline droplets, the concentration of H_2_O_2_ became significantly lower with added CO_2_, implying that in such conditions, the enhancement of OH^–^ leads to the suppression of H_2_O_2_. Among the three different pH values we studied, H_2_O_2_ displayed the greatest sensitivity to the bath gas (with or without CO_2_) only at high pH, where the dissolution of CO_2_ is favored. However, the decrease of H_2_O_2_ cannot be attributed to a decrease of pH due to dissolved CO_2_, as the collected droplets are still alkaline (Figure 1) and the observed H_2_O_2_ is lower than that obtained under neutral and acidic conditions.

The relationship between pH and dissolved CO_2_ is governed by the coupled equilibria (R8) and (R9). Under alkaline conditions, the main carbonaceous species that are present are HCO_3_^–^ and CO_3_^2–^ in a ratio of 0.8, as shown in Figure S6. HCO_3_^–^ can act as an OH radical scavenger via reaction R10 (*k*_10_ = 8.5 × 10^6^ M^–1^ s^–1^).^29^ Although this process is slower than OH recombination by 3 orders of magnitude, based on bulk kinetics parameters, HCO_3_^–^ concentration is expected to be at least 11 orders higher than that of the produced OH radicals, at pH 9.5 (Text S5). Therefore, R10 can be considered as a competitive reaction, minimizing the contribution of OH recombination to oxidant production.
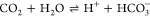
8
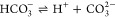
9
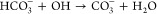
10
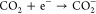
11In addition, CO_2_, prior to its hydrolysis (R8 slow process, *k*_8_≈ 4 × 10^–2^ s^–1^),^30^ may react rapidly with the solvated electrons, via the competitive reaction R11 (*k*_11_ = 7.7 × 10^9^ M^–1^ s^–1^).^31^ Solvated electrons play a key role in the H_2_O_2_ formation mechanism as they react with molecular oxygen to form O_2_^–^. O_2_^–^ reaction with hydronium cations leads to HO_2_ formation, which via R6 and R7 produces H_2_O_2_. Therefore, in the presence of carbon dioxide, fewer electrons are available to react with the dissolved molecular oxygen, resulting in less H_2_O_2_. Both chemical reactions lead to a decrease in H_2_O_2_ formation, in excellent agreement with our results, showing that the reactions initiated by dissolved CO_2_ participate in interfacial H_2_O_2_ production.

To explore further the role of CO_2_, we performed experiments using NaCl solutions of bulk pH 4 and 9.5, varying the concentration of CO_2_ from 0 to 800 ppm. Figure 3 presents the H_2_O_2_ production as a function of the CO_2_ concentration (see Table S2). In acidic conditions, H_2_O_2_ produced at the interface of NaCl droplets is seen to be independent of the CO_2_ content, suggesting that the amount of the dissolved CO_2_ is not sufficient to affect its production. However, under alkaline conditions, H_2_O_2_ displayed a systematic decrease as CO_2_ increases, which highlights the importance of the CO_2_ chemistry and the involvement of reactions R8 to R11.

**Figure 3:**
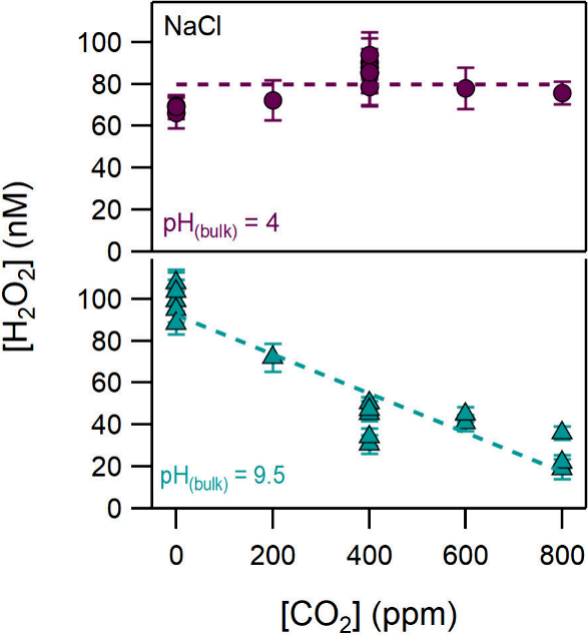
H_2_O_2_ produced in NaCl droplets as a function of the CO_2_ concentration, at bulk pH values of 4 and 9.5.

Finally, Figure 4 displays results of experiments that were performed with NaBr droplets in both UHP N_2_/O_2_ and compressed air environments. Unlike the other salts, in the absence of CO_2_, H_2_O_2_ remained stable, suggesting that the changes in the concentration of the hydroxide anion have a negligible effect on its production. It was recently proposed that bromide anion (Br^–^) may undergo charge separation (Br···e^–^) in the presence of the interfacial electric field,^8,32^ producing Br radicals and electrons (R12).^16^
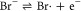
12

**Figure 4:**
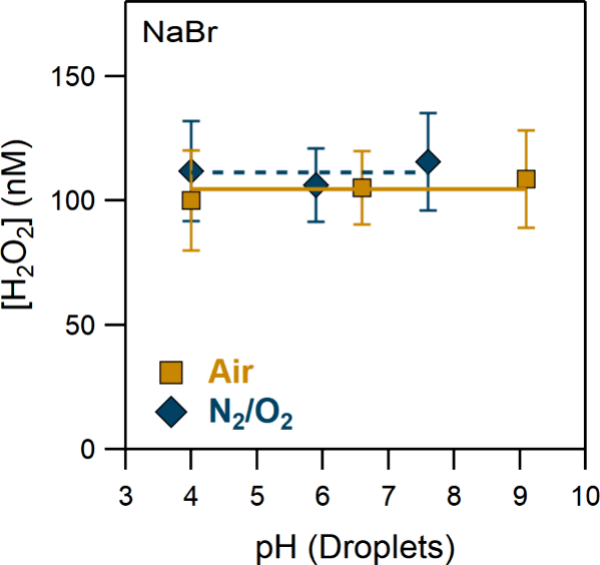
H_2_O_2_, produced in NaBr droplets (pH = 4–9.5), in UHP N_2_/O_2_, and compressed air (see legend).

We suggest that in the presence of a bromide anion the mechanism of H_2_O_2_ formation changes. The enhancement of solvated electrons near the interfacial region of NaBr droplets would promote H_2_O_2_ production via reactions R4-R7, while interfacial OH^–^ (R1) and OH recombination (R3) contribute less to its formation. In addition, no dependence on the bath gas was observed in the range of our measurements. In the presence of CO_2_, electrons can react with both carbon dioxide and oxygen. However, R11 does not seem to have an effect in peroxide production due to the higher electron concentration. The presence of HCO_3_^–^ eliminates further the OH recombination, and thus in both bath gases H_2_O_2_ is mainly formed via HO_2_-initiated reactions (R6-R7).

Overall, these results suggest that the aerosol pH can affect H_2_O_2_ production near the interfacial region. Sea salt aerosols’ pH can be acidic or alkaline, depending on their aging processes and their size. For example, ammonia-enriched environments and high relative humidity will result in almost alkaline droplets, while the uptake of gases such as HNO_3_ and H_2_SO_4_ in sea aerosols will significantly decrease their pH.^18,19^ Therefore, the contribution of interfacial OH/H_2_O_2_ production occurring in marine droplets to the total tropospheric budget will depend on their environment and their composition. The H_2_O_2_ production mechanism seems to be more complex than thought initially. The results given here emphasize the need for further studies to elucidate the concentration of different species at droplet interface and bulk, as well as the inclusion of all of the reactions that are involved in this interfacial process in atmospheric simulation models.

## Supporting Information Available

Text S1 and Figure S1: Description of the experimental procedure, conditions and setup, Figure S2: Size distribution measurements, Text S2 and Figure S3: H_2_O_2_ production as a function of nebulization flow, Text S3 and Figure S4: Bulk H_2_O_2_ measurements as a function of pH, Text S4 and Figure S5: CO_2_ Henry’s law constant as a function of pH, Figure S6: Speciation diagram of CO_2_, HCO_3_^–^ and CO_3_^2–^ as a function of pH. Text S5: Calculations for OH radicals and HCO_3_^–^ concentrations. Table S1: Experimental conditions and results for the H_2_O_2_ production measured in the collected droplets of Na_2_SO_4_, NaCl and NaBr. Table S2: H_2_O_2_ production measured in the collected droplets of NaCl, at pH 4 and 9.5 at different levels of CO_2_ (PDF)
