# Correction to “pH Affects the Spontaneous Formation of H_2_O_2_ at the Air–Water Interfaces”

**DOI:** 10.1021/jacs.4c14211

**Published:** 2024-10-23

**Authors:** Maria Angelaki, Jil d’Erceville, D. James Donaldson, Christian George

**Affiliations:** † Universite Claude Bernard Lyon 1, CNRS, IRCELYON, UMR 5256, Villeurbanne F-69100, France; ‡ Department of Chemistry, University of Toronto, 80 George Street, Toronto, Ontario, Canada M5S 3H6; § Department of Physical and Environmental Sciences, University of Toronto Scarborough, 1265 Military Trail, Toronto, ON, Canada M1C 1A4

d’Erceville’s first name was originally published incorrectly and is now corrected to Jil d’Erceville.

