# Supplementary material for: pH Affects the Spontaneous Formation of H2O2 at the Air–Water Interfaces
Source: J Am Chem Soc. 2024 Sep 16;146(38):25889–93. doi: 10.1021/jacs.4c07356 (PMC11440490; doi:10.1021/jacs.4c07356)
Supplement: Supplementary file 1 — ja4c07356_si_001.pdf [file ja4c07356_si_001.pdf]

pH affects the spontaneous formation of H_2O_2 at the air–water interfaces

Maria Angelaki[1], Jill d'Erceville[1], D. James Donaldson[2,3] and Christian George[*,1]

[1]Université Claude Bernard Lyon 1, CNRS, IRCELYON, UMR 5256, F-69626, Villeurbanne, France

[2]Department of Chemistry, University of Toronto, 80 George Street, Toronto, Ontario, Canada M5S 3H6

[3]Department of Physical and Environmental Sciences, University of Toronto, Scarborough, 1265 Military Trail, Toronto, ON Canada M1C 1A4

[*]To whom correspondence should be addressed: christian.george@ircelyon.univ-lyon1.fr

Keywords: Aqueous droplets, Air- water interface, H_2O_2 formation, Acidity, CO_2 chemistry

Supporting information

Text S1: Experimental description

Figure S1 displays a schematic of the experimental system. Aqueous nanodroplets were generated by nebulizing bulk solutions of 2.5 mM salt concentration at the range of pH 4 – 9.5. All salts were of high purity to avoid possible OH/H_2O_2 generation from trace metals that they may contain[1]: sodium sulfate (Na_2SO_4, suitable for HPLC, 99.0 – 101.0% (T), Merck), sodium chloride (NaCl, ≥ 99%, Sigma Aldrich) and sodium bromide (NaBr, ≥ 99%, Sigma Aldrich). For pH regulation, sulfuric acid (H_2SO_4, 95 – 97%, Merck), hydrochloric acid (HCl, ACS reagent 37%, Sigma Aldrich) and sodium hydroxide (NaOH, 1 N, Merck) were used. All the solutions were prepared by using ultrapure water (Elga Purelab Classic, 18.6 MΩ cm). The pH was measured via a Metrohm pH meter (Model 913)

The nebulization occurred using a commercial glass nebulizer (Meinhard), which consists of two parts. An inner capillary of 0.5 mm, where the water is passing through, and anouter space (5 mm), for the nebulizing gases. Droplets were introduced via an inlet, into a glass flow-tube reactor (V = 1 L) with a flow of 0.25 L min^{-1}. An additional flow of humidified air was added via a second inlet, resulting in a total flow rate of 5.25 L min^{-1}. Taking into account the volume of the reactor and the flow rate, the residence time of the aerosols inside the reactor is estimated to be 10 s. These flows were chosen after performing control experiments, so as to achieve the highest possible sensitivity in our measurements (Text S2 and Figure S3).

The experiments were carried out using air produced via a compressed air generator, or by introducing ultra-high-pure (UHP) N_2 (99.999%) and O_2 (99.999%), in a ratio of 80:20. The compressed air generation system was equipped with air cooler, several dryers and filters for the removal of impurities i.e., water, oil, dirt and particles. The presence of potential impurities and particles was monitored regularly, via PTR–ToF–MS and scanning mobility particle sizers. To minimize the evaporation of the droplets all the gases were humidified by passing through bubblers. All the bubblers were thermostated, so as to avoid changes in humidity due to changes in the temperature, during the experimental procedure. The humidity was constantly monitored inside the reactor via an RH/T probe (VAISALA, HMP110), which was attached in one of the outlets of the reactor. The temperature was also monitored using the same RH/T probe. For all the experiments the humidity and temperature values were 90 ± 2 % and 292 ± 1 K, respectively. To avoid any potential effect of the sunlight in our results, the majority of the experiments were also performed by covering all the parts of the experimental set up with dark optical cloth. The results in the presence and the absence of natural light led to identical results, denoting that no further chemistry can be initiated. At the second outlet of the reactor, an optical particle counter (Palas Promo 2300) was connected, to monitor the optical size distributions of the generated particles. The size of the droplets was between 100 – 700 nm of diameter and it was found to be independent from the pH of the droplets and the type of the salt, in accordance with our previous observations.[2] The size distributions of NaCl droplets, for three different pH, are presented in Figure S2.

The generated droplets were collected in liquid phase at the bottom outlet of the flow-tube reactor in 50 ml glass volumes. The H_2O_2 produced in them was measured off-line, using a H_2O_2 analyzer (Aero-Laser AL-2021). The instrument is of high sensitivity and capable of detecting low concentration of H_2O_2 i.e., 8 nM. A detailed description of the instrument is given in Text S2.

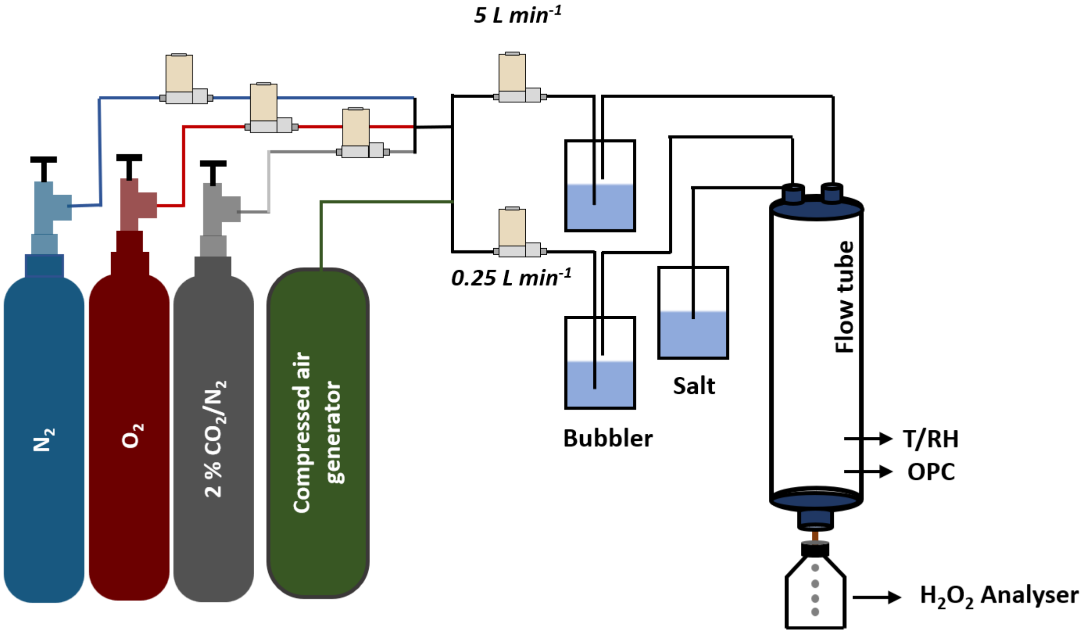

Figure S1. Simplified schematic of the experimental set-up.

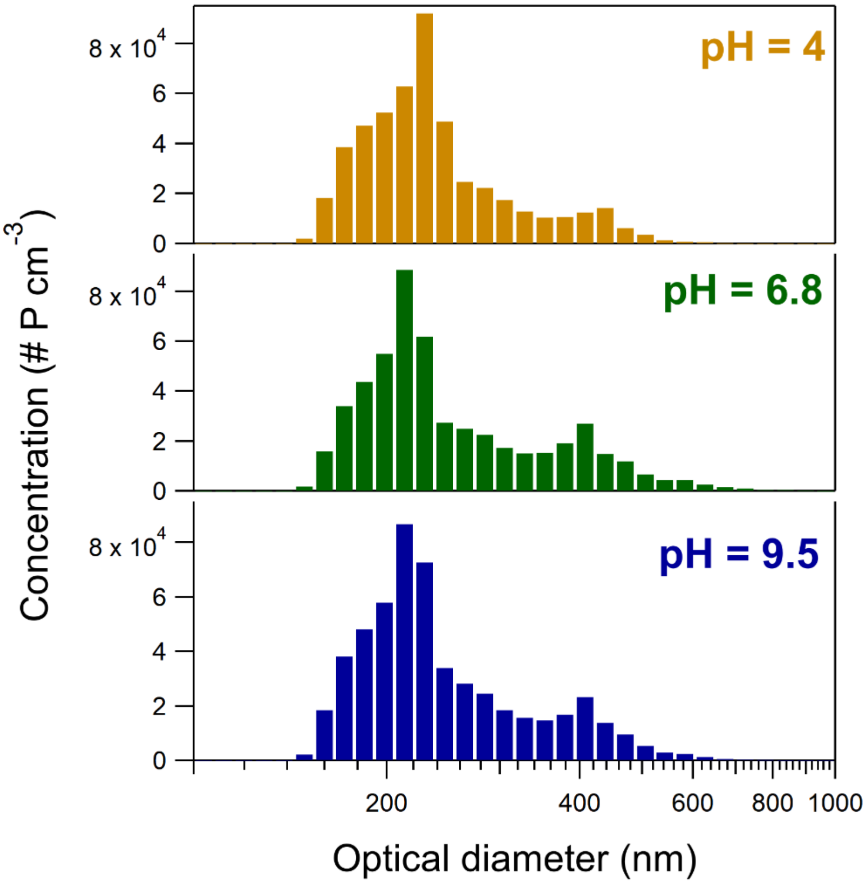

Figure S2. Optical size distributions measured in NaCl particles resulted from the nebulization of bulk solution of pH 4 (top, yellow), 6.8 (middle, green) and 9.5 (bottom, blue).

Text S2: H_2O_2 production as a function of nebulization gas flow.

There are several studies claiming that the interfacial H_2O_2 production depends on the nebulization flow[3,4]. To achieve the highest possible detection sensitivity and also to verify that our results are consistent with previous studies, we performed experiments where H_2O_2 was measured as a function of the nebulization flow rate, at the range of 0.2 to 0.4 ml min^{-1}. The additional flow rate was kept constant, as well as the temperature and the humidity levels. These experiments were performed for NaCl and NaBr droplets. The results are presented in Figure S3. We observed an increase of H_2O_2 with decreasing flow rate, which is attributed to higher droplet lifetimes in the reactor, leading to water evaporation and thus higher H_2O_2 concentrations. The non linear trend that we observed is in accord with the studies of Mehgradi *et al.*,[3] and Mofidfar *et al.*,[4]. However, no significant changes in the size distribution were observed, in contradiction to the observations of Mofidfar *et al.*.[4] For the experiments reported in this work, we chose to nebulize the aqueous solutions with a rate of 0.25 ml min^{-1}, as lower rates significantly decreased the amount of collected droplets, while higher rates led to lower amounts of H_2O_2.

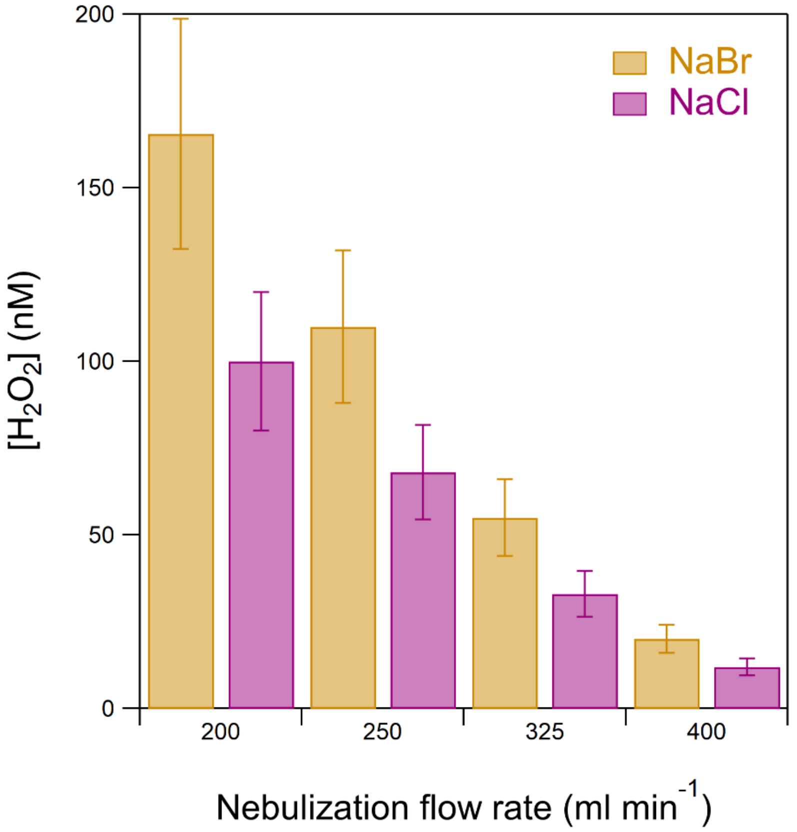

Figure S3. H_2O_2 generated in NaCl (purple) and NaBr (yellow) droplets (initial pH = 6.5), as a function of nebulization flow rate.

Text S3: Bulk H_2O_2 measurements as a function of pH

For the direct H_2O_2 measurements an H_2O_2 analyser was used that detects H_2O_2 via a differential method. The sample is split into two channels, A and B. The two channels contain p-hydroxyphenylacetic acid (POPHA) which reacts with the peroxides to form a fluorescent compound. In channel A, all the peroxides (RO_2 and H_2O_2) are detected, while in channel B, the presence of a catalase (called peroxidase) destroys exclusively the H_2O_2 and allows the detection of the RO_2 via fluorescent spectroscopy. The subtraction of the signals obtained from the two channels gives the H_2O_2 concentration.

Catalase and peroxidase are pH-sensitive, resulting in limitations of the detection, depending on the range of the pH of the measurements. To ensure that the H_2O_2 measurements are not affected by the pH, we performed control experiments in which H_2O_2 bulk solutions of certain concentration i.e., 100 nM were measured at different acidity levels. The results are presented in Figure S4. H_2O_2 concentration remained stable at the expected level over the range of pH 4 to 10, while at pH below 4 and above 10 the signal decreased abruptly as catalase and peroxidase cannot operate in such conditions.

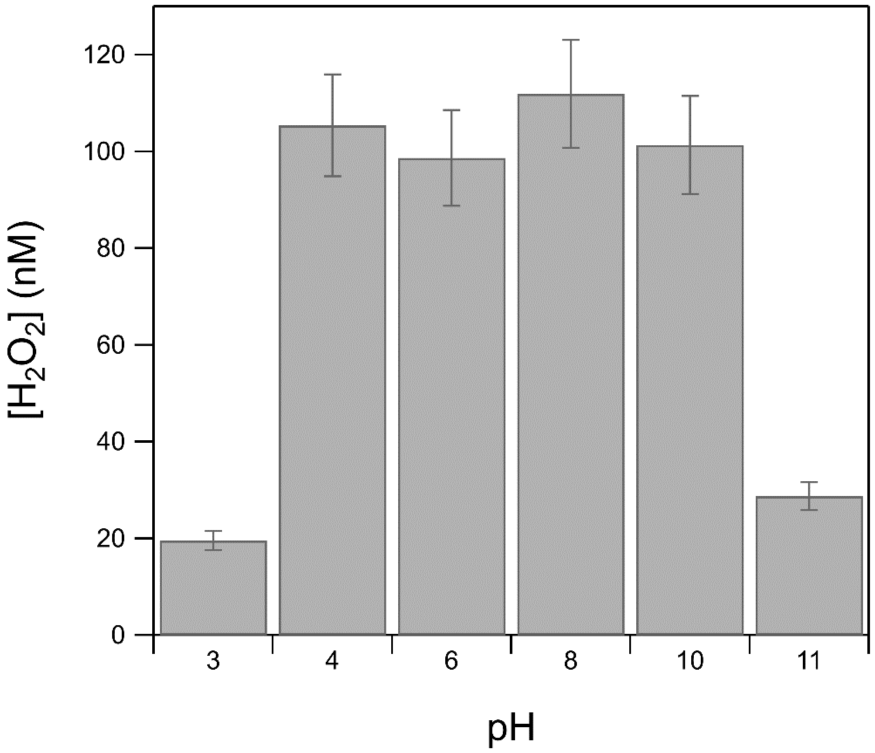

Figure S4. Solution of H_2O_2 of 100 nM concentration measured at different pH.

Text S4: CO_2 Henry's law constant as a function of pH

The Henry's Law constant of CO_2 is well studied in literature, with the value to be $H_{CO2} = 3.4 \times 10^{-2}$ M atm^{-1}.[5] In solution, CO_2 is in equilibrium with H^+ and HCO_3^-, which is also in equilibrium with H^+ and CO_3^{2-} (R1 and R2). Seinfeld and Pandis[6] have suggested that the value of CO_2 Henry's law constant depends on the concentration of hydronium cations through the equation R1.[6] Based on the available literature data, we calculated, through equation R1, the effective CO_2 Henry's law constant, $^*H_{CO2}$, as a function of pH. In Figure S5, the values of H_{CO2} and $^*H_{CO2}$ are presented as dashed line and gray solid symbols, respectively. The yellow symbols present the $^*H_{CO2}$ for the pH conditions in which our experiments occurred. CO_2 dissolution is clearly favoured in alkaline conditions as the Henry coefficient is higher by four orders of magnitude comparing to the acidic environment.

$$CO_2 \cdot H_2O \rightleftharpoons H^+ + HCO_3^-,\ K_{c1} = 4.3 \times 10^{-7}\ \mathrm{M} \quad \text{(R1)}$$

$$HCO_3^- \rightleftharpoons H^+ + CO_3^{2-},\ K_{c2} = 4.7 \times 10^{-11}\ \mathrm{M} \quad \text{(R2)}$$

$$H_{CO2}^{*} = H_{CO2}\left(\frac{K_{C1}}{[H^+]} + \frac{K_{C1}K_{C2}}{[H^+]^2}\right) \quad \text{(Eq1)}$$

From the reaction R1 and R2 and by using the equations Eq2 – Eq4, we also calculated the ratios of the carbonaceous species as a function of pH. The results are presented in Figure S6.

$$\Sigma_{CO_2} = \frac{[H^+]^2}{[H^+]^2 + [H^+]\,K_{C1} + K_{C1}K_{C2}} \quad \text{(Eq2)}$$

$$\Sigma_{HCO_3^-} = \frac{[H^+]\,K_{C1}}{[H^+]^2 + [H^+]\,K_{C1} + K_{C1}K_{C2}} \quad \text{(Eq3)}$$

$$\Sigma_{CO_3^{2-}} = \frac{K_{C1}K_{C2}}{[H^+]^2 + [H^+]\,K_{C1} + K_{C1}K_{C2}} \quad \text{(Eq4)}$$

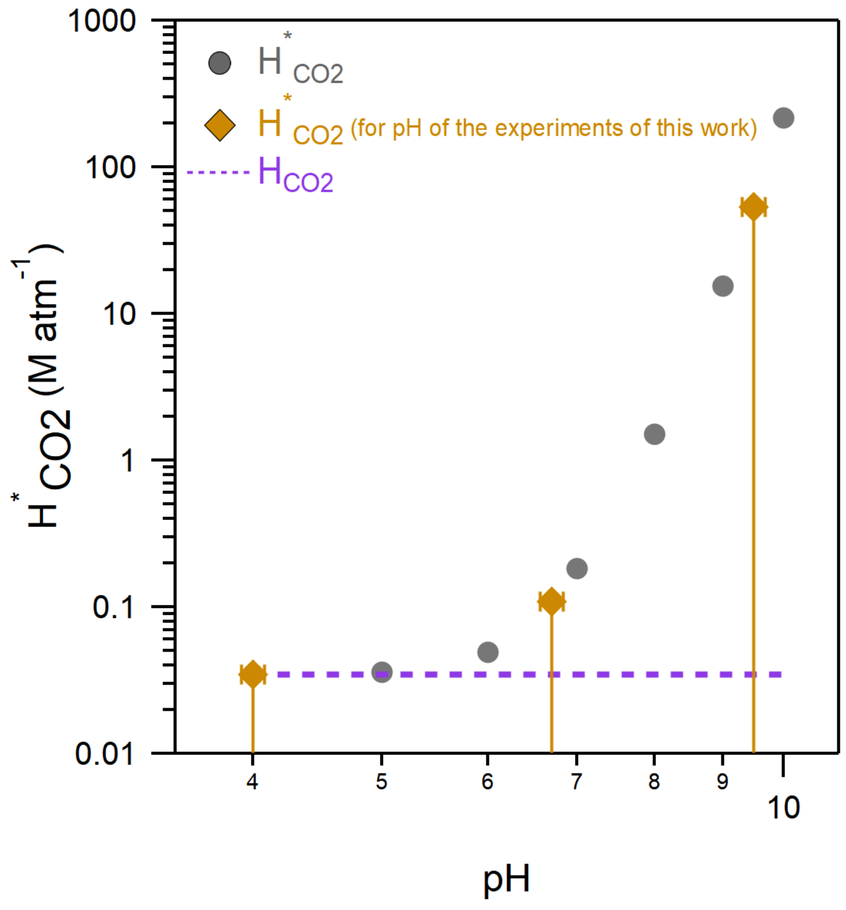

Figure S5. The effective Henry's law constant of CO_2, $*H_{CO2}$, as a function of pH (gray solid circles). Purple dash line: Value of H_{CO2}. Yellow squares: Values of $*H_{CO2}$ for our experimental conditions.

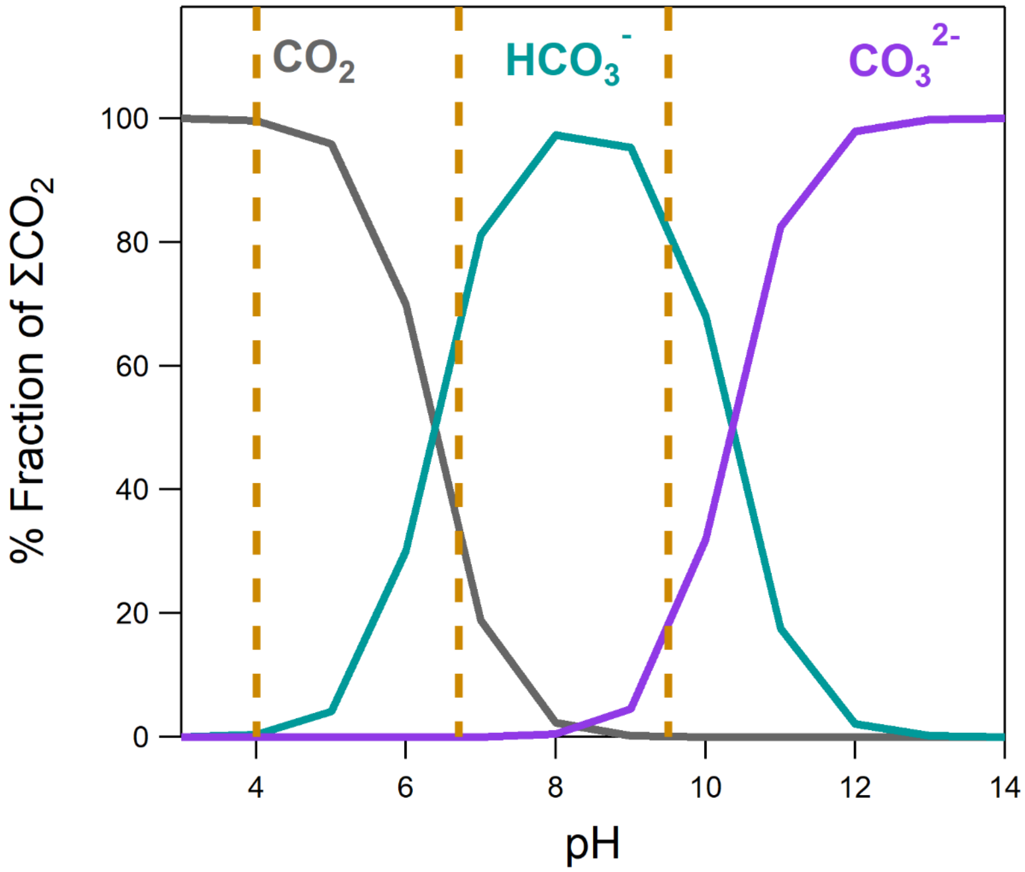

Figure S6. Speciation diagram of CO_2, HCO_3^- and CO_3^{2-} as a function of pH. Yellow dotted lines show the acidity in our experimental conditions.

Text S5: Calculations for OH radicals and HCO_3^- concentrations

In our experiments, H_2O_2, generated in NaCl/Na_2SO_4 droplets and compressed air environment, was measured ~40 nM. Assuming that the sole pathway that leads to H_2O_2 production is the OH radicals recombination, the estimated concentration is [OH] = 2×10^{-10} M. In our previous study, we found that the two main pathways leading to H_2O_2 formation are OH and HO_2 recombination with equal contributions. Considering that, OH concentration is estimated approximately 1×10^{-10} M. HCO_3^- concentration can be calculated by Eq5 and reaction R1. The estimated value for pH 9.5 is $[HCO_3^-] = 2 \times 10^{-2}$ M, while for pH 4 is $[HCO_3^-] = 7 \times 10^{-8}$ M. In both cases, HCO_3^- is much higher than the one of OH, denoting that scavenging of OH by the carbonaceous compound is not negligible.

$$[HCO_3^-] = \frac{[CO_2]\, K_{C1}}{[H^+]} \qquad \text{(Eq5)}$$

Table S1: Experimental conditions and results for the H_2O_2 production measured in the collected droplets of Na_2SO_4, NaCl and NaBr. The colors represent the averaged data for the different bath gases at each pH (blue: UHP N_2/O_2 80:20, yellow: compressed air, purple: UHP N_2/O_2 80:20 + UHP CO_2). These data are presented in Figure 2 and Figure 4.

		$[H_2O_2]$ [b] ± 2σ [c]	
**pH** [a]	**N_2/O_2**	**Compressed air**	**N_2/O_2 + CO_2**
		**Na_2SO_4**	
4	65.88 ± 5.64	86.17 ± 9.54	89.65 ± 17.0
	64.19 ± 5.94	87.64 ± 10.7	
	60.58 ± 7.51	77.9 ± 9.32	
	**63.55 ± 10.5** [d]	**83.90 ± 15.3** [d]	**89.65 ± 17.0** [d]
6.7	63.88 ± 9.51	53.52 ± 7.54	
	65.24 ± 7.43	60.33 ± 5.99	
		69.11 ± 6.82	
		63.54 ± 6.93	
	**64.56 ± 10.2** [d]	**61.62 ± 15.6** [d]	
9.5	97.05 ± 10.2	38.23 ± 3.41	48.23 ± 7.54
	92.65 ± 10.4	40.52 ± 3.42	
	93.13 ± 9.65		
	**94.27 ± 12.3** [d]	**39.37 ± 4.57** [d]	**48.23 ± 7.54** [d]
		**NaCl**	
4	69.54 ± 4.35	90.22 ± 6.22	85.69 ± 16.2
	66.18 ± 7.32	85.12 ± 9.54	94.00 ± 10.8
	69.11 ± 5.69	78.47 ± 8.74	
		87.82 ± 5.68	
	**68.27 ± 9.41** [d]	**85.41 ± 11.0** [d]	**89.84 ± 14.7** [d]
6.7	64.18 ± 7.14	76.05 ± 4.54	
	67.11 ± 7.68	60.35 ± 5.21	
		67.17 ± 3.12	
		58.82 ± 3.69	
	**65.64 ± 8.61** [d]	**65.58 ± 10.7** [d]	
9.5	107.5 ± 6.32	45.29 ± 3.50	33.97 ± 4.11
	99.00 ± 10.2	50.29 ± 2.98	47.20 ± 4.25
	94.92 ± 8.98	30.41 ± 4.56	
	88.24 ± 4.94		
	103.4 ± 9.23		
	**98.64 ± 15.2** [d]	**41.99 ± 12.0** [d]	**40.58 ± 10.7** [d]
		**NaBr**	
4	111.8 ± 20.1	89.41 ± 6.40	
		111.2 ± 8.65	
	**111.8 ± 20.1** [d]	**100.3 ± 20.5** [d]	
6.7	99.1 ± 5.55	105.3 ± 13.8	
	113.6 ± 9.64		
	**106.4 ± 16.6** [d]	**105.3 ± 13.8** [d]	
9.5	115.6 ± 18.1	100.1 ± 12.4	
		116.5 ± 8.12	
	**115.6± 18.1** [d]	**108.3 ± 20.6** [d]	

[a]pH of the bulk solution.

[b]Units of nM.

[c]2σ uncertainties (random and systematic).

[d]The mean value of H_2O_2 concentration for each experimental condition.

Table S2: H_2O_2 production measured in the collected droplets of NaCl, at pH 4 and 9.5 at different levels of CO_2.

**pH** [a]	**[CO_2]** [b]	**[H_2O_2]** [c] **± 2σ** [d]
4	0	69.54 ± 4.35
		66.18 ± 7.32
		69.11 ± 5.69
	200	72.11 ± 9.64
	400	90.22 ± 6.22
		85.12 ± 9.54
		78.47 ± 8.74
		87.82 ± 5.68
		85.69 ± 16.2
		94.00 ± 10.8
	600	78.11 ± 9.90
	800	65.65 ± 5.47
9.5	0	107.5 ± 6.32
		99.00 ± 10.2
		94.92 ± 8.98
		88.24 ± 4.94
		103.4 ± 9.23
	200	72.06 ± 6.68
	400	45.29 ± 3.50
		50.29 ± 2.98
		30.41 ± 4.56
		33.97 ± 4.11
		47.20 ± 4.25
	600	40.88 ± 3.69
		44.71 ± 3.41
	800	18.82 ± 4.65
		22.05 ± 3.65
		36.00 ± 3.22

[a]pH of the bulk solution.

[b]Units of ppm.

[c]Units of nM.

[d]2σ uncertainties (random and systematic).

[d]The mean value of H_2O_2 concentration for each experimental condition.

References

1. Kwan, W. P.; Voelker B. M., Rates of Hydroxyl Radical Generation and Organic Compound Oxidation in Mineral-Catalyzed Fenton-like Systems, *Environ. Sci. Technol.*, **2003**, *37*, 1150-1158.
2. Angelaki, M.; Carreira Mendes Da Silva, Y.; Perrier, S.; George, C., Quantification and Mechanistic Investigation of the Spontaneous H_2O_2 Generation at the Interfaces of Salt-Containing Aqueous Droplets., *J. Am. Chem. Soc.*, **2024**, *146* (12), 8327–8334.
3. Mehrgardi, M. A.; Mofidfar, M.; Zare, R. N., Sprayed Water Microdroplets Are Able to Generate Hydrogen Peroxide Spontaneously. *J. Am. Chem. Soc.* **2022**, *144* (17), 7606–7609.
4. Mofidfar, M.; Mehrgardi, M. A.; Xia, Y.; Zare, R. N., Dependence on Relative Humidity in the Formation of Reactive Oxygen Species in Water Droplets. *Proc. Natl. Acad. Sci. U.S.A.*, **2024**, *121* (12), e2315940121.
5. JPL Publication 19-5. Chemical Kinetics and Photochemical Data for Use in Atmospheric Studies, **2020**.
6. Seinfeld, J. H.; Pandis, S. N., Atmospheric Chemistry and Physics from Air Pollution to Climate Change, John Wiley and Sons, New York, **1998**.
